# Probabilistic generative transformer language models for generative design of molecules

**DOI:** 10.1186/s13321-023-00759-z

**Published:** 2023-09-25

**Authors:** Lai Wei, Nihang Fu, Yuqi Song, Qian Wang, Jianjun Hu

**Affiliations:** 1 Department of Computer Science and Engineering, University of South Carolina, Columbia, SC 29201 USA; 2 Department of Chemistry and Biochemistry, University of South Carolina, Columbia, SC 29201 USA

**Keywords:** Deep learning, Language models, Molecules generator, Molecules discovery, Blank filling

## Abstract

Self-supervised neural language models have recently found wide applications in the generative design of organic molecules and protein sequences as well as representation learning for downstream structure classification and functional prediction. However, most of the existing deep learning models for molecule design usually require a big dataset and have a black-box architecture, which makes it difficult to interpret their design logic. Here we propose the Generative Molecular Transformer (GMTransformer), a probabilistic neural network model for generative design of molecules. Our model is built on the blank filling language model originally developed for text processing, which has demonstrated unique advantages in learning the “molecules grammars” with high-quality generation, interpretability, and data efficiency. Benchmarked on the MOSES datasets, our models achieve high novelty and Scaf compared to other baselines. The probabilistic generation steps have the potential in tinkering with molecule design due to their capability of recommending how to modify existing molecules with explanation, guided by the learned implicit molecule chemistry. The source code and datasets can be accessed freely at https://github.com/usccolumbia/GMTransformer

## Introduction

The discovery of novel organic molecules has wide applications in many fields, such as drug design and catalysis development [1]. However, due to the sophisticated structure–property relationships, traditional rational design approaches have only covered an extremely limited chemical design space [2]. Recently, a large number of generative machine learning algorithms and models have been proposed for molecule design, as systematically reviewed in [1, 3, 4]. The first category of these methods is deep generative models (DGMs), Deep generative models (DGMs) typically leverage deep networks to learn from an input dataset and synthesize new designs [5]. Recently, DGMs such as feed forward neural networks (NNs), generative adversarial networks (GANs) [6], variational autoencoders (VAEs) [7], certain deep reinforcement learning (DRL) frameworks and normalizing flow-based models [8] have shown promising results in design applications like structural optimization, materials design, and shape synthesis. Two of the major limitations of these models include their black-box nature and the challenge of dealing with modularity in molecule design. In [9], Westermayr et al. proposed an approach that combines an autoregressive generative model that predicts three-dimensional conformations of molecules with a supervised deep network model that predicts their properties. The generation of molecules with (multiple) specific properties is achieved by screening newly generated molecules for desirable properties and reusing hit molecules to retrain the generative model with a bias. Despite its efficiency in property-oriented sampling, it lacks interpretability and cannot use modular motifs. Another trend in molecule generation is that explicit 3D molecular generative models have recently emerged [10], aiming to generate molecules directly in 3D, outputting both the atom types and spatial coordinates, either in a one-shot or incrementally adding atoms or fragments. One such model is GeoDiff [11], which is inspired by denoising diffusion generative models for image generation. This model can generate molecular conformations by treating each atom as a particle and learning to directly reverse the diffusion process that transforms from a noise distribution to stable conformations. Flam-Shepherd [12] showed that language models based on LSTM can learn the distributional properties of target datasets using both SMILES and SELFIE representations. It can generate larger, more complex molecules or generate from chemical spaces with large ranges in size and structure, which shows advantages over graph generative models. ORGAN [6] is a GAN based black-box generative model for molecule generation, whose data generation can be subject to a domain-specific reward function. However, its black-box nature makes it difficult to interpret learned logic in terms of the chemical knowledge they learn and how they exploit the learned implicit knowledge for generation. In addition, due to the lack of syntactic and semantic formalization as a limitation of specific structured data, unsuitable generic string generation models often lead to invalid model outputs. We need to prepare a large number of valid combinations of structures in advance to train a reasonable model, which is time-consuming. Although the grammar variational autoencoder (GVAE) [13] directly encodes from and decodes grammar parse trees, aiming to ensure the generated outputs are always syntactically valid, it is still incapable of regularizing the models so that they only generate semantically valid objects.

The second category of molecule generative design methods includes several key combinatorial optimization algorithms such as genetic algorithms [14], reinforcement learning [15], Bayesian optimization [16], Monte Carlo Tree Search (MCTS) [17], Markov Chain Monte Carlo (MCMC) [3]. While GAs have demonstrated superior performance in several molecule design benchmark studies [18, 19], the genetic operators of mutation and cross-over lack the learning capability to achieve intelligent and efficient chemical space exploration. This also applies to MCTS, which locally and randomly searches each branch of intermediates and selects the most promising ones during each generation’s iteration [20]. Bayesian optimization is usually applied together with VAEs and searches the chemical space in the latent space, Jin et al. use Bayesian optimization to optimize molecules generated by a variational autoencoder based on molecular graphs, it generates a tree-structured scaffold over chemical substructures first, and then combines them into a molecule with a graph message passing network [21]. However, the computational complexity of the dimensional space of its search space is relatively high, and its computational complexity increases exponentially with the increase of the dimension of the optimization space, which also makes it difficult to handle the modularity in molecule design. The chemical constraints explicitly [16] are also difficult to achieve. Reinforcement learning has been applied to generative models with both SMILES and 2D graph representations, which learns a policy network to determine the optimal actions that maximize a global reward such as a given property [15, 22]. However, RL is rarely used in de novo molecule generation partially due to the difficulty to achieve long-range credit assignment and to obtain differentiable validity checks as the reward signal.

A pivotal consideration in designing generative models for molecules revolves around the representation level of these molecules, encompassing atom-based, fragment-based, and reaction-based approaches. While the majority of existing models have leaned towards atom-based representations like SMILES, more sophisticated alternatives such as SELFIES [23] and DeepSMILES [24] have emerged for molecule property prediction. The impact of choosing a specific molecule representation on generative design performance remains an unresolved query. Notably, it has been observed that fundamental atom representations, such as SMILES, pose challenges when attempting to harness the modules, motifs, or skeletons present in known molecules. On the contrary, fragment and reaction-based generative models offer the potential to exploit these larger building blocks; however, they also grapple with the intricacies of expressive power.

Another major limitation of existing deep generative models for molecule design is that most of them cannot be used for tinkering design: a specified part of an existing molecule is masked for replacement of other modules to gain specific function property, despite that this is one of the most widely used approaches to explore new molecules [2] due to many constraints imposed on the possible options. During these processes, chemists or molecular scientists usually resort to their intuition, chemical knowledge, and expertise to select substitution or doping elements and proportions to tune the properties of the molecule by considering a variety of factors such as chemical compatibility, poison level, geometric compatibility, synthesizability, and other heuristic knowledge.

Here we propose a self-supervised probabilistic language model, the Generative Molecular Transformer (GMTransformer) for molecular design and generation. The model is based on transformers and the self-supervised blank-filling language model BLM [25]. The model interpretably calculates its probabilities and derives different actions depending on the token frequency shown by its vocabulary. We use SMILES, SELFIES and DeepSMILES representations to train different models, and found that each of them has its own advantage. The easy interpretation, data efficiency, and tinkering design potentials have been demonstrated in our recent work on inorganic materials composition design [26], which inspires us to explore its potential in molecule design in this work. We use MOSES benchmarking metrics to evaluate the performance of our GMTransformer models. The results of our extensive experiments show strong performance compared to state-of-the-art baselines. Our GMTransformer model with SMILES representation achieves 96.83% novelty and 87.01% of IntDiv, which demonstrates that our model is capable of generating a wide variety of novel molecules. We also train generative models for maximizing different properties: logP, tPSA, and QED, and find that our models can learn to generate molecules with specific properties as demonstrated by the distribution of generated molecular properties.^1^

## Methods

### Generative and tinkering molecular design as a blank-filling process

SMILES (Simplified Molecular Input Line Entry System) uses a string of characters to describe describe the connectivity of chemical compounds, focusing on their atomic arrangement and bonding patterns. While SMILES notation effectively captures the structural relationships between atoms and bonds, it’s essential to note that these representations do not convey information about the three-dimensional arrangement of atoms in space. Within SMILES, atoms, bonds, and branches combine to form the strings that represent molecules. The atoms are represented by their element symbols, e.g. C, N, O, S, F. The atoms in aromatic rings are represented by lowercase letters, such as the lowercase c for aromatic carbon. There are three types of bonds in SMILES: single bonds, double bonds, and triple bonds, and they are denoted by -, =, # respectively. Branches are specified by enclosures in parentheses.

**Table 1:** Strings of SMILES generated as a canvas rewriting process

Canvas rewriting with 4 actions: (E, _E, E_, _E_)		
Step t	Action	operation
0. $1	_E_	Replace $1 blank with _C_
1. $1 C $2	E	Replace $1 blank with C
2. C C $1	E_	Replace $1 blank with (_
3. C C ( $1	_E_	Replace $1 blank with _O_
4. C C ( $1 O $2	_E	Replace $1 blank with =
5. C C ( = O $1	E_	Replace $1 blank with )_
6. C C ( = O ) $1	E	Replace $1 blank with C
7. C C ( = O ) C		

As shown in Table 1, the following canvas rewriting process shows how the GMTransformer generates the $$CC(=O)C$$ sequence of the SMILES strings step by step. At the beginning, there is only an initial blank token of $1 on the canvas, then different candidate tokens and rewriting actions (E, _E, E_, _E_) are selected by GMTransformer. (1) action E: replace a blank with the element E; (2) action _E: replace a blank with element E and insert a new blank on its left side, allowing further element insertion; (3) action E_: replace a blank with element E and insert a new blank on its right side, allowing further element insertion; (4) action _E_: replace the blank with element E and insert new blanks on both sides [26]. Finally, a string without any blank symbols is generated on the canvas. In Table 1, there is only one initial blank on the canvas in step 0, and it selects action _E_ with the element C to get $1 C $2. Then it replaces the blank of $1 with the element C by taking action E in the first step. In the second step, the operation replaces $1 blank with a branch (_. Then it chooses action _E_ and replaces the blank with element O. In the next two steps, it replaces the blank with bond = and branch )_ respectively to get canvas C C ( = O ) $1. Finally, it replaces $1 with element C.

GMTransformer is different from BERT [27] and XL-Net [28] as it relies on pre-existing content to learn and generate sequences. Instead of using the context of a pre-masked word to predict the probability of the masked word, GMTransformer directly chooses the action and then inserts the word that best matches the content it learns at the appropriate position based on the probabilistic dependencies in the generated vocabulary.

### Generative molecular transformer: blank filling language model for molecule generation

GMTransformer is not like the black-box models such as Variational Autoencoders (VAEs) [7], Generative Adversarial Networks (GANs) [6], and normalizing flow-based models [8], it is a process interpretable model designed based on a blank language model (BLM) [25]. GMTransformer directly models the probability of the tokens in the vocabulary. It relies on the content of the existing canvas to calculate the probability distribution for selecting actions and tokens to generate a new canvas. It can intelligently control the intermediate process of generating the string, and each step can give an explanation of why it is doing it.

String-based assembly strategies represent molecules as strings and explore chemical space by modifying strings directly: character-by-character, token-by-token, or through more complex transformations based on a specific grammar [29]. SMILES is well-known for its simplicity in denoting molecules as strings by following rules like adjacent atoms are assumed to be connected by a single or aromatic bond and branches are specified in parentheses, etc. In particular, learning valid molecules is substantially more difficult with the SMILES grammar, as there are many more characters to generate for these molecules and a higher probability that the model will make a mistake and produce an invalid string [12]. GMTransformer uses SMILES, SELFIES and DeepSMILES representations of atom-level tokenization. SMILES defines a character string representation of a molecule by performing a depth-first pre-order spanning tree traversal of the molecular graph, generating symbols for each atom, bond, tree-traversal decision, and broken cycles [30]. The SMILES representation of atom-level tokenization has 21 tokens in SMILES strings and 7 special tokens as the vocabulary during the training process. The vocabulary contains 13 atom tokens $<C>,<c>,<O>,<o>,<N>,<n>,<F>,<S>,<s>,<Cl>,, <[nH]>$, and $<[H]>$, 3 bond tokens $<->,<=>, <\#>$, 6 ring tokens $<1>,<2>,<3>,<4>,<5>, <6>$ and 7 special tokens $<PAD>,<UNK>,<FIRST>,<LAST>,<EOS>,<BLANK>, <BLANK\_0>$. SELFIES and DeepSMILES also contain the same 7 special tokens as SMILES.

The SmilesPE tokenization has a mean length of approximately 6 tokens, while the atom-level tokenization has a mean length of approximately 40. SMILES Pair Encoding contains the special tokens and unique tokens from the frequent SMILES substrings. e.g, $<CCC(C)(C)>$, $<CCCC(C)>$, $<NC(=O)C>$. Both SMILES and DEEP SMILES use the SmilesPE tokenization, which does not apply to SELFIES. More details can be found in [31].

**Fig. 1:**
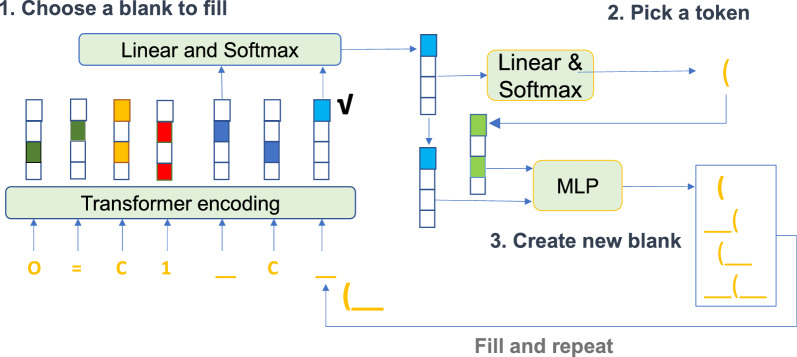
Neural network architecture of the blank filling language model for molecules tinkering using SMILES string $O=C1CC(c2ccccc2)Oc2cc(O)cc(O)c21$ as an example

Figure 1 shows the architecture of our Generative Molecular Transformer (GMTransformer). The model utilizes four networks in three iterative stages. The first stage includes the transformer network and linear and softmax layers. The second and third stages include linear and softmax layers, multi-layer perceptron network, respectively. In the first stage, the transformer network encodes the canvas into a sequence of representations. Then which location of the blank should be filled is selected by computing probabilities from linear and softmax layers. In the second stage, it picks an appropriate token and inserts it into the blank with linear and softmax layers. In the final stage, the action of whether or not to create blanks to the left and right is determined by feeding the concatenation of the representations of the selected blank and the token into the multi-layer perceptron network. The model updates the canvas and repeats the process until there are no blank positions on the canvas.

During the training process, first of all, it initializes the model parameter $$\theta$$ and then randomly samples a training example $$x=\left( x_{1}, \cdots , x_{n}\right)$$ with the length of *n*. Next, it samples the step length *t* from 0 to $$n - 1$$ and the generation order with n-permutation $$\sigma$$ of the given example. It constructs a canvas *c* that remains the first *t* tokens $$x_{\sigma _j}$$ ($$j=1,...,t)$$ and collapses the remaining $$n-t$$ tokens as blanks. Then it takes $$n-t$$ target actions $$a_{j-t}$$ for filling $$x_{\sigma _j}$$ ($$j=t+1,...,n)$$ into canvas and calculates loss as Eq. 1. Finally it updates parameter $$\theta$$ by gradient descent and repeats the whole process until convergence. More details can be found in [25].

1 $$\begin{aligned} -\log (n !)-\frac{n}{n-t} \sum _{\sigma _{t+1}} \log p\left( a_{t}^{x, \sigma } \mid c_{t}^{x, \sigma } ; \theta \right) \end{aligned}$$

where the $\theta$ is the the model parameter; $c_{t}^{x, \sigma }$ is the *t*th canvas with the given training example *x* and the determined generation order (permutation $\sigma$); $a_{t}^{x, \sigma }$ represents the action whether or not to create blanks to the left and right of the predicted token at step *t* with the order permutation $\sigma$ and the selected blank.

### Datasets

We use the dataset from the benchmarking platform Molecular Sets (MOSES) at https://github.com/molecularsets/moses [32]. It contains 1,936,962 molecular structures totally and splits them into three datasets for experiments. Each of them consists of training samples (around 1.6 M), test samples (176 k), and scaffold test samples (176 k) and we use the training and test sets in our experiments. We use the SMILES, SELFIES sets with the basic Atom-level and SmilesPE tokenizers.

### Evaluation criteria

We use the MOSES benchmarking score metrics to evaluate the overall quality of the generated samples. Several models with different tokens are used for GMTransformer training and each model generates 30,000 samples that are evaluated by the MOSES benchmarking metrics in Table 2. The ratios of valid and unique (unique@1*k* and unique@10*k*) report the validity and uniqueness of the generated SMILES string respectively. Novelty is the proportion of molecules in the generated samples that is not in the training set. Filter refers to the proportion of generated molecules that passed the filter during dataset construction. The MOSES metrics also measure the internal diversity (IntDiv) [33], the similarity to the nearest neighbor (SNN) [32], Frechet ChemNet distance (FCD) [34], fragment similarity (Frag) [32], and scaffold similarity (Scaf) [32].

The Internal diversity (IntDiv) is calculated via eq (2), it evaluates the chemical diversity in the generated set *G* of molecules and detects if the generative model has model collapse.

2 $$\begin{aligned} {\text {IntDiv}_{p}}(G) = 1-\root p \of {\frac{1}{|G|^{2}} \sum _{{m_{1}, m_{2}} \in G} \text {T} {\left( m_{1}, m_{2}\right) }^{p}} \end{aligned}$$

Where *G* is the generated set, $m_a$ and $m_b$ are their Morgan fingerprints [35] for two molecules *a* and *b*. *T* is the Tanimoto-distance [36] molecules of generated set *G*.

The Similarity to a nearest neighbor (SNN) is calculated via eq (3).

3 $$\begin{aligned} {\text {SNN}}(G, R)=\frac{1}{|G|} \sum _{m_{G} \in G} \max _{m_{R} \in R} T\left( m_{G}, m_{R}\right) \end{aligned}$$

Where *m* is the Morgan fingerprints of a molecule. T($m_G,m_R$) is an average Tanimoto similarity between $m_G$ in generated set *G* and its nearest neighbor molecule $m_R$ in the reference dataset *R*.

The Fréchet ChemNet distance (FCD) is computed from the activation of the penultimate layer of the deep neural network ChemNet, which was trained to predict the biological activity of drugs. These activations can capture chemical and biological properties of compounds for two sets *G* and *R*. It is defined as Eq. 4):

4 $$\begin{aligned} {\text {FCD}}(G, R)=||\mu _{G}-\mu _{R}||^{2}+{\text {Tr}}\left( \sum _{G}+\sum _{R}-2\left( \sum _{G} \sum _{R}\right) ^{1 / 2}\right) \end{aligned}$$

Where $\mu _{G}$, $\mu _{R}$ are mean vectors for sets *G* and *R* respectively, $\sum {G}$, $\sum {R}$ are full covariance matrices of activations. *Tr* stands for the trace operator.

The Fragment similarity (Frag) is calculated via eq (5), which compares distributions of BRICS fragments [37] in the generated set *G* and reference set *R*.

5 $$\begin{aligned} {\text {Frag}}(G, R)=\frac{\sum _{f \in F}\left( c_{f}(G) \cdot c_{f}(R)\right) }{\sqrt{\sum _{f \in F} c_{f}^{2}(G)} \sqrt{\sum _{f \in F} c_{f}^{2}(R)}} \end{aligned}$$

Where *F* is the set of BRICS fragments. $c_{f}(X)$ stands for the frequency of occurrences of a substructure fragment *f* in the molecules of set *X*.

The Scaffold similarity (Scaff) is similar with Frag but it computes the frequencies of Bemis-Murcko scaffolds [38]. It is calculated as eq (6):

6 $$\begin{aligned} {\text {Scaf}}(G, R)=\frac{\sum _{s \in S}\left( c_{s}(G) \cdot c_{s}(R)\right) }{\sqrt{\sum _{s \in S} c_{s}^{2}(G)} \sqrt{\sum _{s \in S} c_{s}^{2}(R)}} \end{aligned}$$

Where S is the set of Bemis-Murcko scaffolds, $s_{S}(X)$ stands for the frequency of occurrences of a substructure scaffold *s* in the molecules of set *X*.

## Results and discussion

### De novo generative design of molecules composition

#### Training of GMTransformer for hypothetical molecule generation

 We use the MOSES dataset as our benchmark dataset, which is widely used in the generative molecular design community. The performance evaluation criteria is derived from the MOSES package, which is also a standard in generator performance evaluation.

The GMTransformer model was trained and evaluated using the database of the MOSES benchmarking platform. MOSES is a benchmarking platform to standardize the training results of molecule generation models. Its initial dataset, ZINK Clean Leads, contains about 4.6 million molecules. The final dataset was obtained by filtering molecules containing charged atoms (except C, N, S, O, F, Cl, Br, H); macrocyclic molecules with more than 8 molecules in the ring; medical chemistry filters (MCFs) and PAINS filters. MOSES provides both training and test sets and a set of metrics for assessing the quality and diversity of the generated molecules. We also evaluate the generated samples of three additional properties: the octanol-water partition coefficient (logP), the topological Polar Surface Area (tPSA), and the Quantitative Estimate of Drug-likeness (QED) [39] computed from RDKit [40] are used for training the conditional GMTransformer generator.

### Evaluation of GMT’s molecular generation performance

We evaluate the performance of our GMTransformer generators and compare it with that of the reference models using ten evaluation criteria with MOSES metrics including validity, uniqueness (unique@1*k* and unique@10*k*), internal diversity (IntDiv), filters, novelty, the similarity to a nearest neighbor (SNN), Frechet ChemNet distance (FCD), fragment similarity (Frag), and scaffold similarity (Scaf). As shown in Table 2, GMT-SMILES, GMT-PE-SMILES and GMT-SELFIES generate 85.87%, 82.88% and 100% valid samples, respectively. The uniqueness of all models is almost 100%. Especially, the novelty of GMT-SMILES, GMT-PE-SMILES and GMT-SELFIES is as high as 95.31%, 88.29% and 96.83% respectively. At the same time, GMT-SMILES, GMT-PE-SMILES, GMT-SELFIES have the highest values with 85.69%, 85.58%, and 87.01% of IntDiv respectively among all reference models. These high values mean that they can generate samples with higher diversity, which may accelerate the discovery of new chemical structures. For FCD/Test, GMT-PE-SMILES performs best among all models with 19.86%, while GMT-SMILES and GMT-SELFIES have values with72.94% and 377.5%. GMT-SMILES, GMT-PE-SMILES and GMT-SELFIES also achieve high values with 16.50%, 10.87% and 10.96%, respectively.

We also compared our model performance to two recent generative models. In a recent work, Gnaneshwar et al. [41] trained a transformer-based score function (a diffusion model) on SELFIES representations of 1.5 million samples from the ZINC dataset and used the Moses benchmarking framework to evaluate the generated samples on a suite of metrics. The evaluation metrics of validity, Unique@1k and Unique@10k are 100%, 88% and 82%. The performance for the filters, novelty, IntDiv, FCD/Test and FCD/TestSF metrics are 37%, 100%, 90%, 398.4% and 409.2%. We find out that except for IntDiv and novelty, our GMT-PE-SMILES model performs better in terms of all other metrics than this diffusion model with filters of 97.97%, FCD/Test of 75.95% and FCD/TestSF of 19.86%. In the work of Wang et al. [42], the cTransformer method is proposed, which is capable of generating both drug-like compounds (without specified targets) and target-specific compounds. The metrics Valid, Unique@1k and Unique@10k are 98.8%, 100% and 99.9%. For the Frag/Test, Frag/TestSF, SNN/Test and SNN/TestSF, the results are 100%, 99.8%, 61.9% and 57.8%. We find that compared to the cTransformer, our GMT-SELFIES model performance are similar in terms of validity, unique@1k, unique@10k, Frag/Test and Frag/TestSF with 100%, 100%, 100%, 98.69% and 98.31%. Our GMT-PE-SMILES model has relatively lower performance in SNN/Test (57.78%) and SNN/TestSF (54.6%) but it has better interpretability and can be used for tinkering design as described in the Discussion section below.

**Table 2:** Performance comparison of generators using the MOSES Benchmark

			GMT			MOSES reference models			
			GMT- SMILES	GMT-PE- SMILES	GMT- SELFIES	GCT -SGDR	VAE	AAE	char RNN
Validity	$\uparrow$		0.8587	0.8288	**1.000**	0.9916	0.9767± 0.0012	0.9368± 0.0341	0.9748± 0.0264
Unique@1k	$\uparrow$		**1.0000**	**1.0000**	**1.0000**	0.998	**1.0**±**0.0**	**1.0**±**0.0**	**1.0**±**0.0**
Unique@10k	$\uparrow$		0.9998	0.9995	**1.0000**	0.9797	0.9984± 0.0005	0.9973± 0.002	0.9994 ± 0.0003
IntDiv	$\uparrow$		0.8569	0.8558	**0.8701**	0.8458	0.8558± 0.0004	0.8557± 0.0031	0.8562 ± 0.0005
Filters	$\uparrow$		0.9766	0.9797	0.7961	**0.9982**	0.6949± 0.0069	0.9960± 0.0006	0.9943 ± 0.0034
Novelty	$\uparrow$		0.9531	0.8829	**0.9683**	0.6756	0.6949± 0.0069	0.7931± 0.0285	0.8419 ± 0.0509
		Test	0.5381	0.5778	0.4673	**0.6513**	0.6257± 0.0005	0.6081± 0.0043	0.6015 ± 0.0206
SNN	$\uparrow$	TestSF	0.5143	0.5460	0.4485	**0.5990**	0.5783± 0.0008	0.5677± 0.0045	0.5649 ± 0.0142
		Test	0.7294	**0.1986**	3.7750	0.7980	0.0990± 0.0125	0.5555± 0.2033	**0.0732** ± **0.0247**
FCD	$\downarrow$	TestSF	1.2607	0.7595	4.5698	0.9949	0.5670± 0.0338	1.0572± 0.2375	**0.5204** ± **0.0379**
		Test	0.9879	0.9982	0.9869	0.9922	0.9994± 0.0001	0.9910± 0.0051	**0.9998** ± **0.0002**
Frag	$\uparrow$	TestSF	0.9850	0.9958	0.9831	0.8562	**0.9984**± **0.0003**	0.9905± 0.0039	0.9983 ± 0.0003
		Test	0.8661	0.9125	0.8431	0.8562	**0.9386**± **0.0021**	0.9022± 0.0375	0.9242 ± 0.0058
Scaf	$\uparrow$	TestSF	**0.1650**	0.1087	0.1096	0.0551	0.0588± 0.0095	0.0789± 0.009	0.1101 ± 0.0081

Bold value indicates the best performance of samples generated by different models under the same evaluation metric

#### Data efficiency of our GMT model

 We further checked how the amount of training samples affects the generator’s performance. We trained two additional GMT models using 20% and 50% of the SELFIES represented samples and used them to generate 30,000 hypothetical molecules, respectively. We then compared these molecule qualities with those generated by the GMT trained with the whole dataset. The results are shown in Table 3. We find that when we reduced the sample size by 50%, most of the performance metrics only changed slightly. For example, the Frag/Test, Frag/TestSF, IntDiv, IntDiv2, Filters all dropped by less than 0.01, which indicates that we can achieve almost twice the data-efficiency using our model. When we further reduced the training set size to 20%, we found several measures related to diversity and novelty increased while the other performance measures deteriorate, but not too significantly.

**Table 3:** Performance comparison of the GMT models trained with 20%, 50%, and 100% training samples

Training samples		20%	50%	100%
Valid	$\uparrow$	**1.0000**	**1.0000**	**1.0000**
Unique@1000	$\uparrow$	**1.0000**	**1.0000**	**1.0000**
Unique@10000	$\uparrow$	1.0000	0.9998	**1.0000**
FCD/Test	$\downarrow$	4.3961	3.9164	**3.7750**
SNN/Test	$\uparrow$	0.4526	0.4573	**0.4673**
Frag/Test	$\uparrow$	0.9840	0.9850	**0.9869**
Scaf/Test	$\uparrow$	0.8225	0.8049	**0.8431**
FCD/TestSF	$\downarrow$	5.2401	4.7000	**4.5698**
SNN/TestSF	$\uparrow$	0.4362	0.4395	**0.4485**
Frag/TestSF	$\uparrow$	0.9792	0.9802	**0.9831**
Scaf/TestSF	$\uparrow$	0.1340	**0.1461**	0.1096
IntDiv	$\uparrow$	**0.8707**	0.8704	0.8701
IntDiv2	$\uparrow$	**0.8653**	0.8650	0.8646
Filters	$\uparrow$	0.7858	0.7913	**0.7961**
Novelty	$\uparrow$	**0.9790**	0.9751	0.9683

Bold value indicates the best performance of samples generated by different models under the same evaluation metric

#### Interpretability of our GMT model and tinkering design

 First, we demonstrate the interpretability of our GMTransformer models. We selected the SMILES string C C n 1 n n n c 1 S C C ( = O ) N 1 C C c 2 c c c c c 2 1 from the dataset and pre-masked the first token to get a template string <mask> C n 1 n n n c 1 S C C ( = O ) N 1 C C c 2 c c c c c 2 1, then we fed the template string to our model to check the possible substitutions for the masked position. Table 4 shows the predicted substitution tokens sorted by their probabilities. We found that our model correctly predicted the masked token to be carbon with a high probability of 0.895. The next two suggested actions are filling the blank with carbon and adding an additional token on the left or right, both with much lower probabilities (0.048 and 0.046). After inspecting the training samples, we found that there are more than 13 training samples that start with C C n 1 n n n c 1 S C C ( = O ), which establishes a context for making correct probabilistic predictions of the substitutions at the masked positions. This shows that our model successfully learns the statistical dependencies among the different positions of the molecule sequences, which explains their blank-filling suggestions.

**Table 4:** Blank-filling substitution suggestions by GMTransformer explain how its working logic

Substitution token	Probability	Action
C	0.895	0
C	0.048	1
C	0.046	2
=	0.007	3
O	0.002	3
C	0.001	3
=	0	2
O	0	1
N	0	1
(	0	3

The masked template is “<mask> C n 1 n n n c 1 S C C ( = O ) N 1 C C c 2 c c c c c 2 1”

To investigate the capability of our model for tinkering design, we picked a molecule’s SMILES string O = C ( N C c 1 c c c s 1 ) N c 1 c c c ( F ) c c 1 and pre-masked it at 22nd position (F) to get a template string O = C ( N C c 1 c c c s 1 ) N c 1 c c c ( <mask> ) c c 1 and fed it to our GMTransformer model for predicting substitutions at this position. Table 5 shows the probabilities over the candidate tokens and corresponding actions to guide the blank-filling process. We found that our model suggested three substitutions with high probabilities. Besides the masked element fluorine (F) with the probability of 0.297, there are two alternative substitutions with bromine (Br) and chlorine (Cl) for tinkering with this position with the probability of 0.427 and 0.25, respectively. All these three elements belong to the same element group, sharing common chemical properties. which demonstrates that our model has learned to make meaningful tinkering design suggestions based on the learned chemical knowledge.

**Table 5:** Tinkering design based on GMTransformer suggests Br and Cl as replacements for F element

Substitution token	Probability	Action
Br	0.427	0
F	0.297	0
Cl	0.25	0
O	0.019	0
C	0.002	1
S	0.001	1
O	0.001	2
N	0	0
#	0	3
-	0	1

The template string is “O = C ( N C c 1 c c c s 1 ) N c 1 c c c ( <mask> ) c c 1”

#### Process of GMT’s learning of chemical rules

**Fig. 2:**
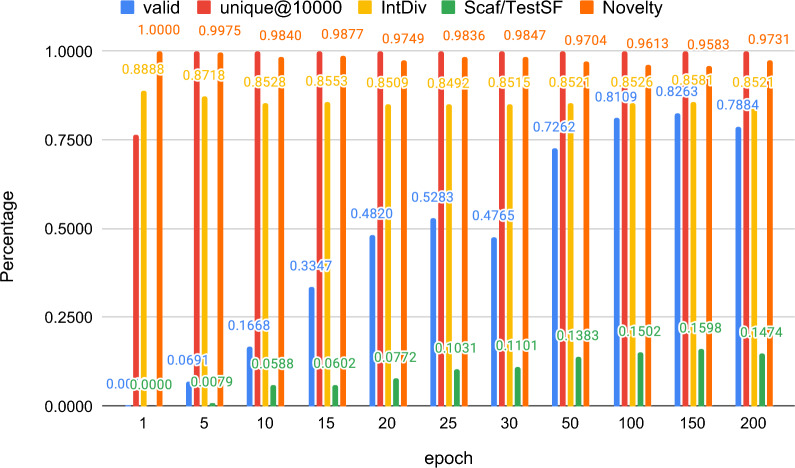
Percentages of valid, unique@10000, intDiv and Scaf/TestSF samples generated by the SMILES atom tokenizer models saved over the training process. The models generate few valid SMILES strings in the beginning. As the training goes on, the models gradually gain the capability to generate chemically valid SMILES molecules compositions

**Fig. 3:**
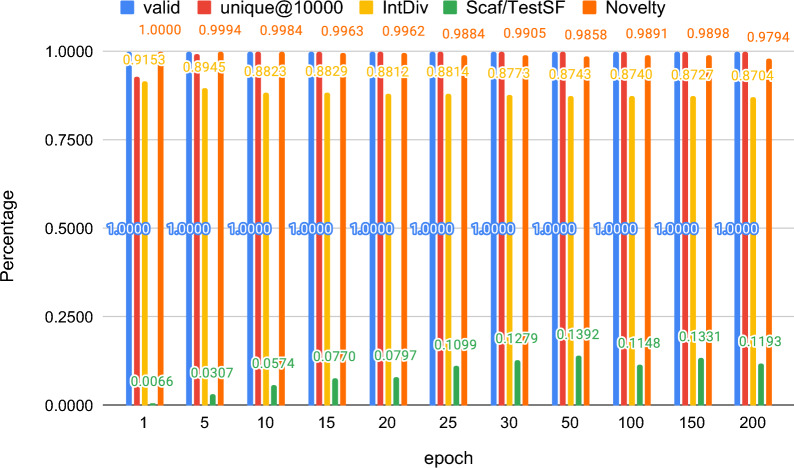
Percentages of valid, unique@10000, intDiv and Scaf/TestSF samples generated by the SELFIES atom tokenizer models saved over the training process. The models generate almost one hundred percent valid SMILES strings from the beginning to the end and the Scaf/TestSF value has also been growing with epoch from 1 to 200

 To illustrate the chemical order/rules emerge during the training process of our GMT models, we save the intermediate models at the end of 1/5/10/15/20/25/30/50/100/150/200 epochs of training using the SMILES and SELFIES dataset, respectively. Then we use 30,000 generated samples to evaluate validity, unique@10k, IntDiv, Scaf/TestSF and Novelty with MOSES benchmarking metrics. As shown in Fig. 2, the validity of the model using the SMILES representation is only about 50% of the maximum value when the epoch of the model training is less than 30, and its validity exceeds 80% at the 100 epochs. This growing process shows that the model is learning the valence rules and the syntax of the SMILES language. For the model using the SELFIES representation, the results are shown in Fig. 3. Because every SELFIES syntax is guaranteed to correspond to a valid molecule [23], the validity is always 100% throughout training epochs from 1 to 200. The increase in Scaf/TestSF value also indicates that the model has learned the Bemis–Murcko scaffold [38], which contains all molecule’s ring structures and linker fragments connecting rings.

### Comparison of different molecule representations: SMILES, SELFIES, and DeepSMILES

Different representations make the model more capable of generating new potential molecules. We use three types of string-based molecular representations: The simplified molecular input line entry system (SMILES) [43], SELF-referencIng Embedded Strings (SELFIES) [44], DeepSMILES [24] and two kinds of tokenizers: Atom-level and SmilesPE [31]. Table 7 shows examples of the different molecule representations with two types of tokenizers. SELFIES only has atom-level tokenizers. We first use SMILES, which is the most widely used representation in computational chemistry. SMILES has some weaknesses such as multiple different SMILES strings can represent the same molecule and it is not robust because it is possible for generative models to create strings that do not represent valid molecular graphs. DeepSMILES is a modification of SMILES which obviates most syntactic errors, while semantic mistakes were still possible [24]. Therefore, we also use the representation of SELFIES, which can generate a 100% effective molecular graph to definitely avoid the problem of model robustness. SELFIES is like an automaton or derivation grammar, which is designed to eliminate syntactic and semantic invalid strings. Atomic-level tokenization is a method commonly used in deep learning, which simply breaks the SMILES string character-by-character, with each character serving as a token. We use not only an atom-level tokenizer but also the SmilesPE representation, which has shorter input sequences and can save the computational cost of model training and inference. SmilesPE identifies and retains frequent SMILES substrings as unique tokens, where each token is represented as a chemically meaningful substructure. We utilize bold and thin strings with spaces between them to distinguish different substrings that are combined into one single tokenizer of SmilesPE in Table 7.

**Table 6:** Performance comparison of GMT models with different representations

			GMT models					
			GMT- SMILES	GMT-QM9- SMILES	GMT-PE- SMILES	GMT- SELFIES	GMT- DEEP	GMT-PE- DEEP
Validity	$\uparrow$		0.8586	0.8937	0.8288	**1.0000**	0.8168	0.7954
Unique@1k	$\uparrow$		**1.0000**	**1.0000**	**1.0000**	**1.0000**	**1.0000**	**1.0000**
Unique@10k	$\uparrow$		0.9998	0.9689	0.9995	**1.0000**	**1.0000**	0.9997
IntDiv	$\uparrow$		0.8569	**0.9182**	0.8558	0.8701	0.8570	0.8519
Filters	$\uparrow$		0.9765	0.6549	0.9797	0.7961	0.9844	**0.9847**
Novelty	$\uparrow$		0.9532	**1.0000**	0.8829	0.9683	0.9367	0.9149
		Test	0.5381	0.2575	**0.5778**	0.4673	0.5509	0.5722
SNN	$\uparrow$	TestSF	0.5143	0.2510	**0.5460**	0.4485	0.5246	0.5405
		Test	0.7294	30.5280	**0.1986**	3.7750	0.3604	0.4366
FCD	$\downarrow$	TestSF	1.2607	31.3022	**0.7595**	4.5698	0.9563	1.0736
		Test	0.9879	0.3945	**0.9982**	0.9869	0.9981	0.9967
Frag	$\uparrow$	TestSF	0.9850	0.3909	0.9958	0.9831	**0.9964**	0.9934
		Test	0.8661	0.0007	**0.9125**	0.8431	0.8880	0.8903
Scaf	$\uparrow$	TestSF	**0.1649**	0.0000	0.1087	0.1096	0.1511	0.1170

Bold value indicates the best performance of samples generated by different models under the same evaluation metric

**Table 7:** Comparison of the different molecule representations: SMILES, SELFIES, and DeepSMILE

Tokenizer	Atom-level
SMILES	C O c 1 c c c c c 1 O C ( = O ) O c 1 c c c c c 1 O C
DeepSMILES	C O c c c c c c 6 O C = O ) O c c c c c c 6 O C
SELFIES	[C] [N] [C] [Branch1] [C] [P] [C] [C] [Ring1] [=Branch1]
Tokenizer	SmilesPE
SMILES	**COc1ccccc1** O **C(=O)O** c1ccccc1 **OC**
DeepSMILES	**CO** cccc **cc** 6 **OC** =O) **O** cccc **cc** 6 **OC**

Bold value indicates the best performance of samples generated by different models under the same evaluation metric

We also train five GMT models using different representations and tokens, generate 30,000 hypothetical molecules and evaluate them using MOSES benchmarking metrics. Table 6 shows the performance of the comparison of the MOSES Benchmarking Results. All models perform very well in terms of uniqueness, in the range of 99.5%-100%. In terms of the novelty of the hypothetical molecules, GMT-PE-SMILES achieves 88.92%, while all other models exceed 90%. GMT-PE-SMILES outperforms the other models by a wide margin on FCD/Test at 19.86%.

We also evaluate our GMT model performance by using the QM9 as the training dataset with the atom-level SMILES representation (Table 7). The validity, unique@1k, unique@10k are 89.37%, 100%, and 96.89% respectively, which are very close to the performance of the GMT trained with MOSES SMILES dataset. The GMT-QM9 is also slightly better in terms of IntDiv and novelty while its filters score is much lower with 0.6549 compared to 0.8569 of GMT-MOSES. Other metrics such as SNN, FCD, Frag, Scaf, they are all evaluated using the reference sets of MOSES, the GMT-QM9 has much lower performance indicating that the training sample distribution strongly affects the properties of generated samples (Table 8).

### Conditional training of generative models for molecule design

**Table 8:** Datasets for conditional generation

	Whole set	Training set (Top 50%)	Generated samples
LogP	1,584,662	792,331	16,748
tPSA	1,584,662	792,331	16,643
QED	1,584,662	792,331	17,082

**Fig. 4:**
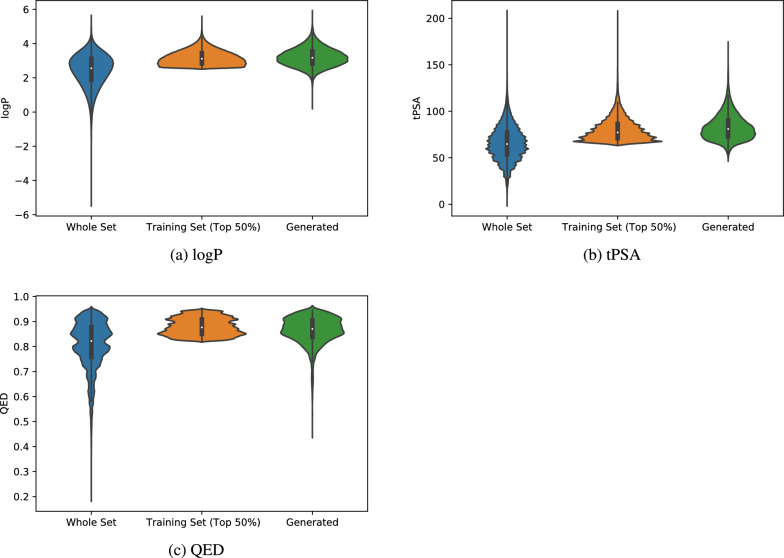
Comparison of property distribution of three different datasets: the whole MOSES training set, the top 50% properties set used for training the conditional generator models, and the generated samples set for logP, tPSA, QED

One desirable generation capability of molecular generators is to design molecules that optimize one or more specific properties. Here we evaluate whether our models have such capability by conditionally training three generators aiming at generating samples with a desired property. This is in contrast to conditional generative models [5] which take the conditions as input. Basically, we prepare three different training sets from the MOSES training dataset by picking samples whose corresponding property values are within the top 50% of the whole MOSES training dataset, where the three properties include the octanol-water partition coefficient (logP), the topological Polar Surface Area (tPSA), and the Quantitative Estimate of Drug-likeness (QED), which are computed using the RDKit. We then train the three generators with these high-property training molecules and use them to generate 20,000 candidate samples, which are then fed to the RDkit for the property calculation. It is found that RDKit cannot calculate the properties for some of these generated samples. After filtering these generated samples, we finally obtain 16,748, 16,643, and 17,082 samples for LogP, tPSA, and QED respectively. The distributions of these properties values of the whole dataset, the biased (top 50%) training set, and the generated candidate sets are shown in Fig. 4. It is found that for all three properties, the distributions of our generated molecules are much closer to those of the top 50% training sets compared to the property distributions of the whole MOSES training dataset, which indicates that the GMTransformer models have learned the implicit rules to generate high-property molecules. It indicates that our models can learn the intrinsic bias of the molecules that are shared among a group of molecules with a desired common property. We can also approach fine-tuning to improve the model’s performance when dealing with a smaller, more focused dataset.

**Fig. 5:**
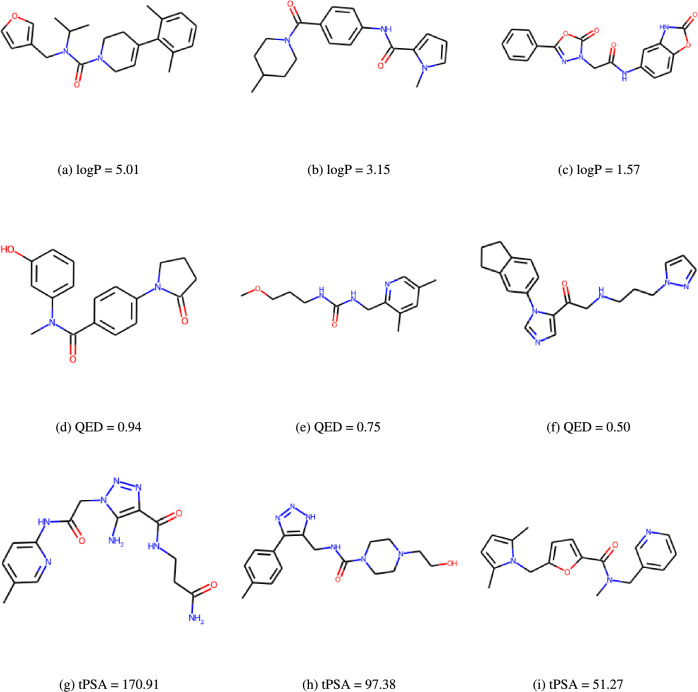
Sample molecular structures generated by conditional training models of logP, QED, and tPSA

In Fig. 5, we present sample molecular structures generated by conditional training models based on properties such as logP, tPSA, and QED. For each property, we selected some of them including the highest score, the lowest score, and an intermediary score for each property to provide a comprehensive view. Figure 5a, b, and c showcase molecular structures generated by the model trained using the top 50% of logP property values. Meanwhile, Fig. 5d, e, and f exhibit molecular structures generated from the model trained with the top 50% of QED property values. Lastly, the structures in Fig. 5g, h, and i are shown from the model trained using the top 50% of tPSA property values.

One possible issue with our conditional training strategy is that we may only have access to a limited number of samples with labeled properties of interest. In that case, we can take the transfer learning strategy: we first train a pre-trained model using datasets with a large amount of labels of related properties. The pre-trained model can then be fine-tuned over the small dataset with the target property labels.

### Discussion

The ability to generate new potential molecular structures has broad implications for a variety of fields, including drug discovery, materials science, and renewable energy. It has the potential to revolutionize the development of new drugs, lead to the creation of new materials with desirable properties, and help advance the development of renewable energy technologies. This ability has the potential to drive innovation and advance our understanding of the world around us, with significant implications for the future of science and technology. It is of benefit to incorporate synthesis knowledge into computational approaches such as small molecule de novo design in order to enhance the practical relevance of the results and achieve better acceptance by medicinal chemists [45].

While uniqueness, validity, and novelty are evaluated mainly based on the molecule structure, the relevance of generated samples to druggability and biological processes is not clear. To address this issue, we evaluate our models using the FCD criterion [34], which is computed using the activation of the penultimate layer of ChemNet. This criterion can capture both the chemical and biological property of the generated molecules. We find that out of the six language models (in Tables 2 and 6), our GMT-PE-SMILES achieves the best performance in terms of the FCD/Test measure with 19.86%, while GMT-SMILES shows the performance with 72.94% and the baseline GCT-SGDR shows 79.80% of the FCD/Test. However, the FCD/Test performance of the GMT-SELFIES model is relatively low without a clear reason. We also find the FCD performance is also relatively low in other relevant models [41, 46] that also use SELFIES representation.

To evaluate how the hyper-parameters may affect the model performance, we utilize SMILES representations with Atom-level tokenizer for hyper-parameter tuning. We use 5, 10, 15, and 20 transformer layers to train the model, then generate 30,000 samples and evaluate the criteria with the MOSES benchmarking metrics. As shown in Table 9, the overall performance of the metrics is similar for each model, the best of which is when the number of layers is 15. The values of Validity, Unique@10000, Filters, and Novelty at this point are 86.46%, 99.99%, 98.06%, 94.13% respectively. The values of FCD/Test and Scaf/TestSF are 32.08% and 15.41% respectively. We use the default number of layers for the model of 6 instead of 15 because hyper-parameter studies show that the number of layers has little effect on the overall performance of the model, and the model with the default number of layers has higher efficiency.

In addition to the ability to generate molecular structures from scratch, our model has a potential application: molecular optimization. This feature consists of shielding specific portions of a given molecular structure and then utilizing our model to intelligently complete those shielded portions. Unlike the traditional method of regenerating molecules, our model optimally adapts the existing structure. We have applied this method in materials [26] with good results. With this approach, we can extend the utility of our model to a wider range of molecular design tasks. This improvement makes our model an important tool for a variety of applications in the molecular design process. From generating new structures to refining existing ones, our model demonstrates its multifaceted potential in improving the efficacy and efficiency of molecular design strategies.

**Table 9:** Hyper-parameter tuning of GMTransform molecules generator

Number of layers		5	10	15	20
Valid		0.8582	0.8488	0.8646	0.8549
Unique@1000		1.0000	1.0000	1.0000	1.0000
Unique@10000		1.0000	0.9997	0.9999	0.9998
IntDiv		0.8529	0.8536	0.8541	0.8540
Filters		0.9802	0.9838	0.9806	0.9812
Novelty		0.9351	0.9389	0.9413	0.9362
SNN	Test	0.5559	0.5556	0.5509	0.5554
	TestSF	0.5277	0.5279	0.5252	0.5304
FCD	Test	0.5404	0.3243	0.3108	0.3903
	TestSF	1.1415	0.8461	0.7978	0.8609
Frag	Test	0.9939	0.9950	0.9965	0.9966
	TestSF	0.9904	0.9913	0.9933	0.9950
Scaf	Test	0.8954	0.8902	0.8955	0.8868
	TestSF	0.1425	0.1482	0.1541	0.1285

## Conclusion

We propose the Generative Molecular Transformer (GMT), a probabilistic generative language model based on neural networks and transformers for the generation and design of molecules. Our model is based on the blank filling language model, which has a unique advantage and potential for tinkering molecule design as we showed in both in this study (Tables 4 and 5) ans well as in our previous work for tinkering design of materials compositions [26]. Since there are many design constraints in real-world molecule and drug design, most of the time, the tinkering design is a preferred approach which starts from an exciting molecule and then finetunes its structures. Our GMT model thus conveniently provides a way for such tinkering design. The advantages of the GMT model also include its interpretability and data efficiency as shown in this study (Table 3) as well as in our previous work on generating hypothetical inorganic materials [26]. Overall, we have shown that our probabilistic transformer model can efficiently learn the grammar rules of molecules and exploit them for generating high-quality hypothetical molecules.

Another advantage of our GMTransformer for molecule generation is that it allows the use functional groups of molecules as tokens to train models that generate molecules with specific functions. The advantage over a simple substructure search for the respective functional group for linking is the ability to directly construct the virtual product. Changes introduced by replacing a part of the structure can thus be scored in the context of the complete molecule [45]. While fragment-based models have been proposed before, the blank filling model we use here can be used to discover those function groups as highly dependent subsequences. The discovery and usage of these special functional groups of molecules may have great potential for molecule design for specific functions suitable for real-life scenarios [47]. For example, fragment-based design has unique advantages in drug design [48]. We also find that the molecule sequence rewriting probabilities and interpretability of the GMT model provide more control over the molecular generation process, which brings more potential for generating molecules with specific properties. This has been demonstrated in our materials composition design using the BLM model [26]. We believe that data efficiency, interpretability, and modularity are three key features that are required for next-generation generative molecule design algorithms.

## Data Availability

he raw molecules QM9 dataset is downloaded from http://quantum-machine.org/datasets/. The modified code can be found at http://github.com/usccolumbia/GMTransformer

## Footnotes

1 Citation: L.W...J.H. Generative transformer for generative molecules design. DOI:000000/11111.
